# An assessment of survey measures used across key epidemiologic studies of United States Gulf War I Era Veterans

**DOI:** 10.1186/1476-069X-12-4

**Published:** 2013-01-09

**Authors:** Rebecca B McNeil, Catherine M Thomas, Steven S Coughlin, Elizabeth Hauser, Grant D Huang, Karen M Goldstein, Marcus R Johnson, Tyra Dunn-Thomas, Dawn T Provenzale

**Affiliations:** 1 Durham Epidemiologic Research and Information Center, Durham Veterans Affairs Medical Center, Durham, NC, USA; 2 Department of Epidemiology, Rollins School of Public Health, Emory University, Atlanta, GA, USA; 3 Department of Medicine, Duke University, Durham, NC, USA; 4 Cooperative Studies Program, Office of Research and Development, Department of Veterans Affairs, Washington, DC, USA; 5 Durham Veterans Affairs Medical Center, Durham, NC, USA; 6 Health Services Research and Development, Durham Veterans Affairs Medical Center, Durham, NC, USA; 7 Clinical Informatics, Raleigh, NC, USA

**Keywords:** Persian Gulf War 1991, Survey methods, Blood banks

## Abstract

Over the past two decades, 12 large epidemiologic studies and 2 registries have focused on U.S. veterans of the 1990–1991 Gulf War Era. We conducted a review of these studies’ research tools to identify existing gaps and overlaps of efforts to date, and to advance development of the next generation of Gulf War Era survey tools. Overall, we found that many of the studies used similar instruments. Questions regarding exposures were more similar across studies than other domains, while neurocognitive and psychological tools were the most variable. Many studies focused on self-reported survey results, with a range of validation practices. However, physical exams, biomedical assessments, and specimen storage were not common. This review suggests that while research may be able to pool data from past surveys, future surveys need to consider how their design can yield data comparable with previous surveys. Additionally, data that incorporate recent technologies in specimen and genetic analyses would greatly enhance such survey data. When combined with existing data on deployment-related exposures and post-deployment health conditions, longitudinal follow-up of existing studies within this collaborative framework could represent an important step toward improving the health of veterans.

## Background

Approximately 697,000 members of the U.S. Armed Forces were deployed to Southwest Asia in support of the 1990–1991 Gulf War. More than 4 million remaining troops were deployed elsewhere or not deployed. Elevated rates of measurable symptomatology and illness in Gulf War Era veterans have been well documented [1-4], with the excess burden of symptom prevalence estimated to be as high as 25-30% among deployed Gulf War troops, while their disease-related mortality remains equivalent to, or even lower than, that of the general population [1-6]. Many epidemiologic studies have been performed to understand the physical and psychological symptoms observed in and reported by veterans who served during this conflict era [1,2,4,7-17]. However, a clear pathologic explanation or overarching diagnosis has remained elusive. While studies initially focused on post-deployment mental health, the number of reports of medically unexplained chronic multisymptom illness in this veteran population increased during the 1990s [18,19]. This attracted the attention of researchers in several disciplines, including immunology, toxicology, neurology, occupational (military) health, epidemiology, and environmental health. Consequently, assessments in research expanded to address physical health, symptoms, and deployment-related exposures. Relative to epidemiological research in most other clinical areas, Gulf War-related studies have involved a wide diversity of outcome measures, risk factors, cohort characteristics, and methods. Novel strategies will be required to unify and harmonize the approach to both clinical care and research for this population in the setting of such broad clinical outcomes and diverse risk factors.

A deeper understanding of the tools used by past studies will enable critical evaluation and direction for future efforts. The Institute of Medicine and the Research Advisory Committee on Gulf War Veterans’ Illnesses have both completed comprehensive reviews of Gulf War research [20-23]. However, these focused primarily on the findings of the many studies conducted thus far, and not the research tools used to gather the studies’ data. There has not been a recent systematic review of the broad range of assessments used in Gulf War research. In this manuscript, we describe and review the research tools used by the fourteen major Gulf War Era studies to date. It is our intent that this information will assist ongoing research efforts through the following two focused purposes: to provide an overview of the assessment tools used in past studies, and to discuss how future Gulf War research may benefit from consideration of commonalities and gaps in the epidemiologic research.

### Inclusion and exclusion criteria

We used three primary criteria to select candidate studies for inclusion in this review. First, we included epidemiologic studies of U.S. veterans that were considered “major cohorts” by the Institute of Medicine’s (IOM) 2009 Gulf War and Health review [22], according to their specified criteria for methodologic rigor, health outcomes assessment, medical evaluations, and use of laboratory testing. Second, we included two Gulf War veteran registries established by the Departments of Veterans Affairs and Defense; while these necessarily suffer from the limitations common to registries, such as non-generalizability and absence of a control group, they jointly comprise a vast repository of standardized data that may be useful in future carefully constructed research efforts. Finally, we considered studies reported since the IOM’s 2009 review, and ongoing studies.

We excluded studies of non-U.S. veterans and clinical studies or trials related to specific disease entities. Substudies of primary studies were included under the umbrella of their original study.

These criteria resulted in a total of twelve epidemiologic studies and two large government registries. The specific studies are: Devens Cohort Study (Devens, originally the Ft. Devens Operation Desert Storm Reunion Survey) [7,24], New Orleans study [8], National Survey of Gulf War Era Veterans and Their Families (National Health Survey) [10], Pennsylvania-Hawaii study [17], Iowa study [2], Air National Guard study [4], Navy Seabees study [12], Oregon-Washington study [13], cross-sectional Kansas study (Kansas I) [1], case–control Kansas study (Kansas II) [14], Millennium Cohort Study [15], Military Health Survey [16], and the VA Persian Gulf War Health Examination Registry (VA Registry) [9] and DoD Comprehensive Clinical Evaluation Program Registry (DoD Registry) [11]. A tabulation of study characteristics is contained in Table 1. Demographic characteristics of the study participants are summarized in Table 2.

**Table 1:** Design characteristics of major studies of U.S. veterans of the first Gulf War

**Study**	**Inception**	**Design**	**Administration**	**Sample**	**Focus**	**Survey Pilot Testing**	**References**
**Devens Cohort Study**	1991	Longitudinal cohort, substudies	In-person surveys and evaluations, mailed surveys	84 units returning from GW through Ft. Devens, MA	Psychological health, domestic and military exposures	Unable to determine^a^	[7,25-27]
**New Orleans**	1991	Longitudinal cohort, substudies	In-person surveys and evaluations, mailed surveys, phone	Units based in Louisiana	Psychological and physical health, stressors	Unable to determine^a^	[8]
**VA Registry**	1992	Registry, substudies	In-person survey and two-stage health evaluation	Self-nominated veterans of 1st GW and OIF	Physical health, military exposures	Unable to determine^a^	[28]
**Nat’l Health Survey**	1992	Longitudinal cohort, substudies	In-person evaluations, mailed surveys, phone surveys	Stratified random sample of GW-deployed and non-deployed	Physical and psychological health, military exposures	Reviewed by OMB	[10,29]
**PA & HI**	1993	Cross-sectional	In-person distribution	All active duty, National Guard, and reserve units in PA and HI	Physical and psychological health	Unable to determine^a^; similar survey used in previous studies	[17]
**DoD Registry**	1994	Registry, substudies	In-person survey and two-stage health evaluation	Self-nominated veterans of 1st GW and OIF	Physical health, military exposures	Unable to determine^a^	[30]
**Iowa**	1995	Cross-sectional, substudies	In-person health evaluation, phone survey	Stratified random sample of GW regular military and NG/Reserve from Iowa	Physical and psychological health, functional status, military exposures	Tested in 24 veterans from the random sample, and 3 non-sampled members of the military	[2]
**Seabees**	1997	Cross-sectional	Mailed survey, phone survey	Construction Battalion (CB) members who served for 30+ days active duty during GW	Current and past health issues, exposures, behavioral risk factors	Tested in Navy personnel, reviewed by OMB and DoD, test-retest reliability on earlier survey	[12,31]
**OR & WA**	1995	Cross-sectional, case–control	In-person health evaluation, mailed survey, phone survey	Random sample of veterans from OR or WA who were deployed to SW Asia between 8/1/90 and 7/31/91	Physical and psychological health, military exposures	Test-retest reliability survey (exposures) in 305 case–control study participants	[32]
**Air Nat’l Guard**	1994	Cross-sectional, substudies	In-person health evaluation and surveys	GW veterans from a PA-based Air National Guard unit, two AF reserve units (PA, FL), and an active duty AF unit (FL)	Physical health, risk factors for illness	Unable to determine^a^	[4,33]
**Kansas I**	1998	Cross-sectional	Phone survey	KS veterans or reserve members who served on active duty between 8/90 and 7/91	Physical health, military exposures	Health questions tested in MO veterans	[1]
**Kansas II**	2000	Case–control	In-person survey and blood draw, phone	Veteran KS/MO residents of Kansas City metropolis, who deployed to GW between 8/1/1990 and 7/31/1991, with Gulf War Illness (cases) or not ill (controls)	Gulf War Illness, military exposures	Tested in veterans residing outside of sampling frame	[14]
**Millennium Cohort**	2000	Longitudinal cohort	Mailed survey, web survey	Stratified random sample of regular active duty, NG, and reserve	Physical health (chronic illness), risk factors	Focus groups and 1000-participant pilot survey (total 2564 tests)	[34]
**Military Health Survey**	2007	Cross-sectional, substudies	In-person blood sample, phone survey	Stratified random sample of deployed and deployable nondeployed between 8/2/90 and 7/31/91	Physical health, military exposures, behavioral risk factors	200-veteran sample pilot tested survey content, interview procedures	[16,35,36]

Abbreviations: *AF *Air Force, *DoD *Department of Defense, *FL* Florida, *GW *Gulf War, *HI *Hawaii, *KS *Kansas, *MA *Massachusetts, *MO *Missouri, *NG *National Guard, *OIF *Operation Iraqi Freedom, *OMB *Office of Management and Budget, *OR *Oregon, *PA *Pennsylvania, *SW *southwest, *VA* Department of Veterans Affairs, *WA *Washington.

^a^We were unable to determine from published sources whether the investigators conducted pilot testing or early validation testing of their survey instruments; however, this was likely done in many instances.

**Table 2:** **Characteristics of participants in major studies of U.S. veterans of the first Gulf War**^a^

		**Devens Cohort Study**	**New Orleans**	**VA Registry**	**Nat’l Health Survey**	**PA & HI**	**DoD Registry**	**Iowa**	**Seabees**	**OR & WA**	**Air Nat’l Guard**	**Kansas I**	**Kansas II**	**Millennium Cohort**^**c**^	**Military Health Survey**	**Total**
**Sample size**		2,949^b^	1,520^d^	66,227	20,917	4,344	54,244	3,695	11,868	1,119	3,723	2,030	304	45,372	8,020	226,332
**Deployment status**	GW	2,345	1,520	66,227	11,441	1,524	46,625	1,896	3,831	1,119	1,154	1,548	304	9,248	5,699	154,481
	Non-GW				9,476	2,727	4,888	1,799	8,037		2,569	482		36,124	1, 192	67,294
**Age, years**	<25							1,895								1,895
	17-21			15,008								386				15,394
	22-25			14,887								426				15,313
	>25							1,800								1,800
	26-31			14,390										5,761		20,151
	>31			21,947										41,471		63,418
	26-33											528		10,118		10,646
	>34											690		35,254		35,944
	Mean	30.2	29		31		32.5		30.6	26.4	35			39.1		
**Date of age**		1991	deploy	deploy	1991			8/1990	1990	deploy	1995	1/1991		2000		
**Gender**	Male	2,137	1,307	59,697	16,715		47,301	3,360	11,334	962	3,202	1,766	282	35,460		183,523
	Female	208	213	6,530	4,202		6,943	335	534	157	521	264	22	9,895		29,824
**Race**	White	1,975	867	43,182	15,550	3,078	27,936	3,543	10,432	1,041	3,202	1,786	265	31,988		144,845
	Black	176	456	16,527	3,828		15,893		843			162	26	6,501		44,412
	Hispanic	84	182	1,752			2,712					61	15	2,319		7,125
	Other	110	15	4,766	1,539	977	7,648	152	593	78	521	41	8	4,537		20,985
**Service**	Active	265		42,590	8,082	2,291	44,697	1,953	10,266	772	1,680	914		21,516		135,026
	NG/Res			23,637				1,742		347		1,116				26,842
	NG	1,494			5,759						1,205			10,863		19,321
	Reserve	586			7,076	1,714	6,455		1,602		838			12,954		31,225
**Branch**	Army	2,345		47,850	13,238^d^		43,504	2,083		526		1,238	168	20,325		131,277
	Air Force			4,148	2,693		3,634	532		67	3,723	447	28	14,945		30,217
	Marines			8,977	2,304		2,658	503		213		142	58	1,569		16,424
	Navy			5,252	2,682		2,712		11,868	313		223	49	7,986		31,085
	Other						1,682	577						547		2,806
**Rank**	Enlisted	982	1,398	61,797	17,553	3,581		3,298	10,244		3,202	1,705	241	326		104,327
	Officer	155	122		3,060			397	1,624			325	63			5,746
	Noncom	1,208												32,219		33,427
	Com			3,686										11,537		15,223
	Warrant			744	288									1,290		2,322
**Reference**		[7]	[8]	[37]	[10]	[17]	[11]	[38]	[12]	[13]	[4]	[1]	[14]	[15]	[16]	

Abbreviations: *Com *Commissioned Officer, *DoD *Department of Defense, *GW *Gulf War, *HI *Hawaii, *NG *National Guard, *Noncom *Noncommissioned Officer, *OR *Oregon, *PA* Pennsylvania, *Res *Reserve Service, *VA *Department of Veterans Affairs, *WA *Washington.

^a^Some cells may not total to the full study sample size due to rounding imprecision in published percentages, or missing data or errors in the original publication.

^b^Figures presented are based on 2,345 participants of Devens Cohort Study, as reported in [7]. Figures for the full cohort of 2,949 are not available in published literature.

^c^Figures presented are based on the subset of Millennium Cohort participants who served during the era of the first Gulf War. These are unpublished data that were provided to us in a personal communication by Dr. Nancy Crum-Cianflone of the Naval Health Research Center.

^d^Denotes that the figure is reported variably in multiple publications, or is otherwise uncertain.

### Survey instruments

The major domains surveyed by the studies and registries are summarized in Tables 3, 4, and 5. These comprise the domains of mental and physical health (Tables 3 and 4) and deployment-related exposures (Table 5). These areas are commonly assessed during epidemiological research on military cohorts.

**Table 3:** Health domains surveyed by major studies of U.S. veterans of the first Gulf War

**Domain**	**Devens Cohort Study**	**New Orleans**	**VA Registry**	**Nat’l Health Survey**	**DoD Registry**	**PA & HI**	**Iowa**	**Seabees**	**OR & WA**	**Air Nat’l Guard**	**Kansas I**	**Kansas II**	**Millennium Cohort**	**Military Health Survey**
**Medical history and clinical diagnoses**	X, X_S_	X_S_	X, X_S_	X	X		X, X_S_	X, X_S_	X	X	X	X	X	X
**Symptoms**	X, X_S_	X, X_S_	X, X_S_	X	X	X	X	X, X_S_	X	X	X	X	X	X
**Functional and health status**	X, X_S_	X_S_	X, X_S_	X, X_S_			X	X	X_S_	X_S_	X	X	X	X
**Fatigue, CFS, fibromyalgia, MCS, MSI**	X, X_S_		X_S_	X, X_S_		X	X	X, X_S_	X_S_	X_S_	X	X	X, X_S_	X
**PTSD**	X, X_S_	X, X_S_	X_S_	X, X_S_	X_s_	X	X, X_S_	X, X_S_	X_S_	X_S_	X	X	X	X
**Clinical evaluation and validation**	X_s_		X, X_S_	X_S_	X		X, X_S_	X_S_	X, X_S_	X_S_		X	X	X_S_
**Family and social support**	X	X_S_					X_S_							
**Intelligence**	X_S_	X_S_		X_S_			X_S_							
**Personality**	X	X_S_	X_S_											
**Psychiatric status**	X, X_S_	X, X_S_	X_S_	X_S_	X_S_	X	X	X_S_	X_S_				X	X
**Depression**		X, X_S_		X, X_S_	X_S_		X		X_S_	X_S_			X	X
**Anxiety**		X, X_S_		X_S_			X		X_S_				X	X
**Memory**	X_S_	X_S_		X_S_			X_S_		X_S_					X
**Executive function**	X_S_	X_S_	X_S_	X_S_			X, X_S_	X	X_S_					
**Psychomotor function**	X_S_	X_S_		X_S_			X_S_	X_S_		X_S_				
**Quality of life**				X_S_									X	
**References**	[3,7,25,26,39-43]	[3,8,42,44-47]	[9,24,48-52]	[10,29,53-56]	[30,57]	[17,58,59]	[2,60,61]	[12,31]	[32,62,63]	[4]	[1]	[14]	[34,64,65]^a^	[16,35]

Abbreviations: *CFS *chronic fatigue syndrome, *DoD *Department of Defense, *HI *Hawaii, *MCS *multiple chemical sensitivity, *MSI *multisymptom illness, *OR *Oregon, *PA *Pennsylvania, *PTSD* post-traumatic stress disorder, *VA *Department of Veterans Affairs, *WA *Washington.

Note: subscripted “S” designates that some data for that domain are only available for a subset of the study sample.

^a^ Additional reference: personal communication by Dr. Nancy Crum-Cianflone of the Naval Health Research Center.

**Table 4:** Psychological status evaluations used in major studies of U.S. veterans of the first Gulf War

**Psychological Domain**	**Instrument**	**Acronym**	**Reference**
Family and social support	Social Provision Scale		[66,67]
	Family Relationship Index	FRI	[68]
	Social Support Questionnaire		[69]
	Social Support		[70]
	Family Stress		[71]
	Family Adaptability and Cohesion Evaluation Scale	FACES-II	[72]
	Relationship Quality		[73]
Intelligence	National Adult Reading Test	NART	[74]
	Wechsler Adult Intelligence Scale	WAIS	[75]
	Shipley Institute of Living Scale	SILS	[76,77]
Personality	Neuroticism, Extroversion, Openness Personality Inventory	NEO-PI	[78]
	Dispositional Resilience Scale	DRS	[79]
	Eysenck Personality Questionnaire		[80]
Psychiatric status	Hopkins Symptom Checklist	HSCL	[81,82]
	Minnesota Multiphasic Personality Inventory-2	MMPI-2	[83]
	Personality Assessment Inventory		[84]
	Composite International Diagnostic Interview	CIDI	[85]
	Symptom Checklist-90-Revised	SCL-90R	[86]
	Diagnostic Interview Schedule	DIS	[87]
	Brief Symptom Inventory	BSI	[88,89]
	Structured Clinical Interview for DSM-III-R	SCID	[90]
	Health Screening System	HSS	[91]
Depression	Prime-MD/Patient Health Questionnaire	PHQ	[92,93]
	Beck Depression Inventory	BDI	[94]
Anxiety	Anxiety Sensitivity Index	ASI	[95]
	Beck Anxiety Inventory	BAI	[96,97]
	State-Trait Anxiety Inventory		[98]
Cognitive Functioning	Digit Span		[75]
	California Verbal Learning Test	CVLT	[99]
	Rey Auditory Verbal Learning Test		[100,101]
	Test of Memory Malingering	TOMM	[102]
	Wechsler Memory Scale	WMS	[103,104]
	Recognition Memory Test		[105]
	Heaton Memory Test		[106]
	Continuous Visual Memory Test		[107]
	Auditory Verbal Learning Test	AVLT	[108]
	Trail-Making Tests A & B		[109,110]
	Continuous Performance Test		[111,112]
	Cognitive Failure Questionnaire		[113]
	Wisconsin Card-Sorting Test		[114]
	Stroop Test		[115]
	Performance On-line		[116]
	Rey-Osterrieth Complex Figure Test		[117,118]
	Paced Auditory Serial Addition Test	PASAT	[119]
	Controlled Oral Word Association Test	COWAT	[120]
	Neurobehavioral Evaluation System Continuous Performance Test		[121]
	Behavioral Assessment and Research System	BARS	[122]
	Symbol Digit		[123]
	Simple Reaction Time		[124]
	Oregon Dual-Task Procedure		[125]
Psychomotor function	Grip Strength Test		
	Symbol Digit		[123]
	Grooved Pegboard		[126]
	Purdue Pegboard		[127]
	Finger Tapping Test		[109,128]
Health perception	Sickness Impact Profile		[129,130]
	Barsky Amplification Scale		[131]
	Illness Behavior Questionnaire		[132]
Quality of Life	Quality of Life Inventory		[133]

**Table 5:** Deployment-related exposure domains surveyed by major studies of U.S. veterans of the first Gulf War

**Exposure**	**Devens Cohort Study**	**New Orleans**	**VA Registry**	**Nat’l Health Survey**	**PA & HI**	**DoD Registry**	**Iowa**	**Seabees**	**OR & WA**	**Air Nat’l Guard**	**Kansas I**	**Kansas II**	**Millennium Cohort**	**Military Health Survey**
**Vaccinations**														
Any vaccine	X						X			X	X	X		X
Anthrax	X		X, X_S_	X		X		X	X				X	X
Typhoid			X_S_	X				X						
Botulism			X, X_S_	X		X		X	X					X
Immune globulin			X_S_	X				X						X
Plague			X_S_	X				X						X
Meningococcus			X_S_	X				X						
**Medications**														
Malaria pills			X_S_	X		X		X_S_						
Pyridostigmine bromide	X	X_S_	X, X_S_	X		X	X	X, X_S_	X	X		X		X
Ciprofloxacin & antibiotics	X		X_S_	X				X, X_S_						
**Airborne exposures**														
Wore chemical protective gear	X		X_S_	X	X		X	X					X	X
Petrol fuels/solvents or fumes			X	X		X	X	X_S_	X					X
Smoke, oil fires, combustion products	X	X_S_	X, X_S_	X	X	X	X	X, X_S_	X	X		X		
Smoke from tent heaters	X	X_S_	X, X_S_	X		X	X	X, X_S_	X					X
Vehicle exhaust	X	X_S_		X										
Chemical or biological warfare agents	X	X_S_	X_S_					X_S_	X	X			X	X
Nerve gas			X	X		X	X							
Mustard gas			X	X		X	X							
SCUD missile or artillery explosions nearby; debris contact	X	X_S_	X_S_	X		X	X		X	X		X		X
Burning trash or feces	X	X_S_	X, X_S_	X			X		X				X_S_	
CARC paint			X	X		X	X		X			X		X
**Radiation sources**														
Depleted uranium			X	X		X	X	X_S_	X			X	X	X
Microwaves			X	X		X	X						X	
**Sources of infection or contaminants**														
Contaminated or local food	X		X	X		X	X	X	X	X				
Contaminated or local water	X	X_S_	X	X		X	X	X	X	X				
Pesticides	X	X_S_	X	X		X	X	X	X	X		X	X	X
Live/dead animals or insects	X		X_S_	X			X	X	X			X		
**Psychological trauma**														
Life events	X		X_S_	X	X		X		X_S_	X			X	
Combat-related stressors	X	X	X_S_	X_S_		X	X		X_S_				X	X
Direct combat duty	X		X, X_S_	X	X	X	X	X		X		X	X	X
Witnessed casualties	X		X_S_	X	X	X	X	X		X		X	X	X
Contact with POW			X_S_	X			X	X				X	X	X
Physical injury	X		X_S_		X	X	X			X			X_S_	X
Suffered forced sexual relations, assault, or sexual harassment			X_S_	X			X		X_S_				X	X
**Behavioral risk factors**														
Alcohol use	X	X_S_	X_S_	X	X		X	X	X	X			X	X
Tobacco use	X	X_S_	X	X	X	X	X	X	X	X		X	X	X
**References**	[3,25-27,40,134]	[3,8,45,135]	[48,51]	[10,136,137]	[17,58,59]	[30,138-140]	[2,38]	[12,31]	[13,32,141]	[4,33,142]	[1]	[14]	[34,143-145]^a^	[35]

Abbreviations: *CARC *Chemical agent resistant coating, *DoD* Department of Defense, *HI *Hawaii, *OR *Oregon, *PA* Pennsylvania, *POW *Prisoner of war, *VA *Department of Veterans Affairs, *WA *Washington.

Note: subscripted “S” designates that some data for that domain are only available for a subset of the study sample.

^a^Additional reference: personal communication by Dr. Nancy Crum-Cianflone of the Naval Health Research Center.

As described in further detail below, the domains of mental and physical health include health status, functional status, symptoms, fatigue complex (fatigue, chronic fatigue syndrome (CFS), fibromyalgia, and multiple chemical sensitivities), post-traumatic stress disorder (PTSD), neurocognitive and/or psychological status, and clinical evaluations and validations. The domains of environmental exposures include vaccinations, medications, airborne exposures, radiation sources, sources of infection or contaminants, psychological trauma, and behavioral risk factors. Each of these exposure domains has multiple subdomains. This review is not a comprehensive representation of all areas of research in these studies, but includes the domains most frequently studied thus far, that are arguably perceived to be the most critical.

### Mental and physical health domains

#### Medical history and clinical diagnoses

Comparable survey measures were used by multiple studies. Eight studies (Devens, Kansas I, Kansas II, National Health Survey, Millennium Cohort, Seabees, Military Health Study, and a VA Registry sub-study) asked whether participants had been diagnosed or treated by a clinician for any of several medical conditions. There was substantial overlap in the conditions queried, including diabetes, depression, asthma, bronchitis, and chronic fatigue syndrome [1,9,12,14,24,25,29,34,35,146]. It was also common to ask the approximate onset date for each condition. In contrast, Oregon-Washington and the VA and DoD registries had open-ended questions about health history, which resulted in a broad range of responses that are not easily comparable [9,30,48,62].

Some instruments were used by only one study. For example, Iowa alone drew questions from instruments such as the National Health Interview Survey [147], the Behavioral Risk Factor Surveillance Survey [148], and the Agricultural Health Study [2,149,150].

### Symptoms

Symptoms were assessed by all of the studies. They were typically queried by asking if the veteran had experienced persistent or recurring symptoms during the 12 months prior to the survey, using a symptom checklist. The number of symptoms surveyed varied by study (8 to 78 symptoms). In addition, some studies collected information about symptom severity and date of onset [1,3,4,10,12,14,16,17,24,29,30,34,35,48,60,62,136,151-153]. New Orleans [8] and Devens [25] evaluated symptoms using the Health Symptom Checklist [81,82]. Devens also used a variant of the Psychosomatic Complaint Scale (Psychological Well-Being Scale) [26,79,154,155]. In later survey versions, Devens used the Expanded Health Symptom Checklist, which included additional questions on symptom presence during the past 30 days, duration, trajectory, and frequency [3,25,26]. National Health Survey used the 15-item Patient Health Questionnaire to measure the occurrence of somatic symptoms during the 4 weeks prior to survey administration [29,156].

In this subdomain, the registries and Iowa differed the most from the rest of the studies. The registries both used open-ended questions to assess symptoms, instead of a binary format [9,30,48]. Iowa asked additional questions about symptoms of asthma and bronchitis from the American Thoracic Society Questionnaire [157,158].

### Functional status and health status

Of the eight studies that evaluated functional status, all used the SF-12, SF-36, or Veterans SF-36, either alone or in combination [2-4,16,29,34,39,53,63,159-163]. The only study that asked questions to further evaluate functional status was the Iowa study, which administered the Health Utilities Index-Mark 3 [164,165].

Health status was one of the most commonly assessed domains. All of the studies, with the exception of Pennsylvania-Hawaii, administered a health status evaluation. However, few of the instruments were directly comparable because their response scales varied, making it difficult to compare the responses. For instance, the Military Health, National Health Survey, Air National Guard, Kansas II, VA Registry, and Seabees all asked a general health rating question [4,9,12,14,24,29,35,48,153]. However, National Health Survey, Air National Guard, and the VA Registry used a five-category response scale, while Kansas II used a four-category response scale, and the Seabees used a three-category response scale. In addition, the response scale anchor points used by Air National Guard and National Health Survey differed from that used by the VA Registry, and the responses cannot be directly compared.

### Chronic multisymptom illness and related diagnoses

It is estimated that chronic multisymptom illness and other symptom-based diagnoses affect up to 25 to 32% of the Gulf War veteran population [23]. Although several instruments have been developed to diagnose these conditions, they are still challenging to distinguish due to the number of their symptoms that overlap with other illnesses. Eleven of the studies collected data on at least one of these illnesses: fatigue, chronic fatigue syndrome, fibromyalgia, multiple chemical sensitivity, and multi-symptom illness.

Fatigue was assessed by Iowa, Devens, and a VA Registry substudy using the Chalder Fatigue Scale [2,60,166], the Fatigue Severity Scale [40,167], and the Multidimensional Fatigue Inventory [49,168], respectively. Chronic fatigue syndrome was most commonly identified in National Health Survey, Iowa, VA Registry, Air National Guard, Kansas II, Military Health Study, a Devens substudy, and Seabees [2,4,12,14,24,29,31,35,41,53,169] using the Centers for Disease Control definition developed by Fukuda [169]. In addition, a National Health Survey substudy validated self-reported chronic fatigue syndrome, fibromyalgia, and other conditions by physician examination [53]. Fibromyalgia was evaluated by four studies (Iowa, a National Health Survey substudy, Military Health Study, and Oregon-Washington) using the American College of Rheumatology criteria [2,35,53,62,170,171]. Multiple chemical sensitivity was queried in five studies, but they each used different measures. These measures included the Chemical Odor Intolerance Index and a scale developed by the Agency for Toxic Substances and Disease Registry [35,49,50,172,173] and multiple internally-developed measures [12,25,40,41,174]. Several studies inferred the presence of medically unexplained multisymptom illness based on the symptoms and medical conditions that they surveyed [1,4,12,14,17,24,35,41,175,176]; however, the 2005 National Health Survey asked questions specifically about participants’ experiences with unexplained multisymptom illness. These included years of first and most recent experience, activities and treatments that improve or aggravate the condition, and status relative to initial diagnosis [29,153]. In addition to questions about chronic fatigue syndrome, fibromyalgia, and multiple chemical sensitivity, the Military Health Study contained a large number of self-constructed questions about illness symptoms in support of their goal of validating a case definition for Gulf War Illness [16,35].

### Post-traumatic stress disorder (PTSD)

Combat-related stress was initially hypothesized to be one of the factors responsible for the illnesses afflicting Gulf War veterans [177]; as a result, it was one of the few factors surveyed by all of the studies. The most frequently used instruments included the Clinician Administered PTSD Scale (4 studies) [30,42,48,54,178,179], PTSD Checklist Military and Civilian versions (6 studies) [2,8,25,26,29,40,60,63,64,180,181], Mississippi Scale for Combat-Related PTSD (3 studies) [61,63,182], Mississippi Scale for Desert Storm War Zone Personnel (3 studies) [4,8,42,183], the Structured Clinical Interview for DSM-III-R - Patient Edition (3 studies) [30,44,48,90,184], and the Impact of Events Scale (2 studies) [51,58,185]. Additional PTSD instruments, including the Penn Inventory for PTSD, were used by individual studies [63].

### Psychological status

Psychological status is a very broad subdomain and is by far one of the most discordant areas in assessment across studies. It includes several fields, such as family and social support, intelligence, personality, psychiatric status, depression, anxiety, memory, executive function, psychomotor function, health perception, and quality of life. There were a total of 59 tests used in this domain, and all of the studies except Kansas I and Kansas II evaluated at least some of the fields. Many of the survey instruments and tests were only used by one study, but the most frequently used instruments were the Wechsler Adult Intelligence Scale and the Trail-Making Tests, which were each used by 5 studies to measure intelligence and executive function, respectively. A summary of the instruments used to evaluate psychological status can be found in Table 4.

### Clinical evaluations and stored biospecimens

Several of the studies have performed detailed clinical evaluations, including complete physicals with standard laboratory tests (VA and DoD Registries, Iowa, Air National Guard, Oregon-Washington, and a National Health Survey substudy) [4,9,13,24,30,53,165]. Both registries have implemented a battery of additional laboratory tests for veterans requiring further evaluation [9,30,52]. The Devens and Military Health studies have performed MRIs [16,35,43]. Many of the studies have performed analyses using blood specimens, particularly for clinical laboratory measures; however, for most, it is not clear whether sufficient samples remain to support additional biomarker research. To our knowledge, only Seabees, Military Health Study, and Kansas II have collected biospecimens for future studies [14,16,31,35,186].

### Healthcare expenditures and utilization

Veterans of this era varied in their approaches to utilizing healthcare services, and this variability was noted both within and between deployed and non-deployed veterans. In particular, participation in health registries that involved a clinical evaluation, such as the VA and DOD registries, accounted for a significant number of medical encounters. Health expenditures were directly addressed only by Iowa, using the National Health Interview Survey and the National Medical Expenditure Survey [2,187-189]. Iowa also administered a telephone survey on health utilization to a subsample of their population who suffered from multiple chemical sensitivity [190]. Devens and National Health Survey used rough measures of health utilization, asking the number of clinic visits or hospitalizations [10,26].

### Deployment-related exposure domains

#### Vaccinations

Receipt of vaccinations prior to or during deployment was assessed by all but two studies (New Orleans and Pennsylvania-Hawaii). The vaccinations queried included anthrax, botulinum, typhoid, meningococcus, plague, a generic “any vaccinations,” and receipt of immune globulin. The form and scope of the questions can be divided roughly into two categories: those that asked whether any vaccines had been received (Iowa, Kansas I, Kansas II, Air National Guard, and Military Health Study) [1,2,14,33,35], and those that asked if specific vaccines had been received (Devens, VA and DoD Registries, National Health Survey, Seabees, Oregon-Washington, Millennium Cohort, and Military Health Study) [10,12,15,25,30,32,35,40,48,51,136].

#### Medications

Three medications were commonly assessed: malaria prophylaxis (any), ciprofloxacin and/or other antibiotics, and pyridostigmine bromide [2,3,10,12-14,31,35,48,51,134,138,142]. Use of these medications was queried using a binary format, or a categorical format that could be collapsed to binary. However, it was common to attempt to elicit additional details of pyridostigmine bromide use, in the form of the total number of pills used, frequency of use, number of days on which a certain dose was exceeded, and the occurrence of specified side effects or feelings of illness after using the pills [2,13,35,51,134,136]. Some studies also asked open-ended questions about the use of over-the-counter and prescription medications [13,35].

#### Airborne exposures

Many of the Gulf War studies assessed exposure to airborne toxins. These contaminants included petroleum fuels, solvents, fumes, smoke, combustion products from oil fires or incinerated trash/feces, tent heater smoke, vehicle exhaust, chemical agent resistant compound paint, debris from missile or artillery explosions, and chemical or biological warfare agents.

All of the studies except Kansas I asked questions regarding airborne exposures. These exposures were most commonly assessed using a binary response or a categorical format that could be collapsed to binary. In addition, several studies asked additional questions about the number of days or times exposed to airborne toxins; these types of questions had either open-ended or categorical response types [3,10,12-14,17,25-27,31,35,38,48,51,58,136,138,142,143]. In addition, Iowa asked whether the exposure was temporally associated with feelings of illness [38].

#### Radiation sources

The most commonly surveyed sources of radiation were depleted uranium and microwaves. Most of the studies queried these exposures using a binary or a categorical format that could be collapsed to binary [10,13,31,38,48,138,144]. In addition, National Health Survey and Iowa both asked about time period of exposure [38,136].

Military Health Study and Kansas II evaluated depleted uranium exposure using questions regarding contact with destroyed enemy vehicles (or inhalation of smoke from vehicles) that may have been struck with artillery rounds containing depleted uranium [14], and exposure to the Camp Doha fire. They also asked whether participants had undergone a urine test for the presence of depleted uranium, the timing and provider of the test, and the results [35].

#### Sources of infection or contaminants

Many of the studies surveyed their participants regarding exposure to potential sources of infection or contamination, which included food, water, pesticides, and local fauna. The response formats for most of the questions were at least binary [10,12-14,25,32,33,35,38,48,51,136,138,142]. Similar question phrasing was used for National Health Survey, Iowa, and the VA Registry [38,48,136] and furthermore, National Health Survey and Iowa used the same three-point scale to describe the length of exposure [38,136]. Devens and a New Orleans subset also used identical tools to evaluate exposures [3,25,45]. Additional exposure information was gathered in three studies. A VA registry subset was asked about the frequency of each exposure’s occurrence [51]. Devens inquired about water and pesticide use, including source of drinking water, unusual smell or tastes in the water, any illnesses or health problems caused by the drinking water, pesticide name, who sprayed the pesticide, presence of any acute symptoms, and exposure frequency [25]. Military Health Study also asked questions about frequency and quantity of application [35].

#### Psychological trauma

The experiencing of psychologically stressful events was one of the primary factors surveyed in the initial studies of veterans of the Gulf War. The perceived importance of this factor is demonstrated by its inclusion in all of the studies except Kansas I. The most commonly studied items were life events and combat-related stressors, the latter of which included direct combat duty, witnessing of casualties, POW contact, physical injury, and the experience of sexual harassment and/or sexual assault or rape.

Because combat-related traumatic events have long been recognized for their effects on psychological health, some tools for their assessment existed at the initiation of the Gulf War studies. Instruments used in these studies included the Combat Exposure Scale [191,192], the Mississippi Scale (Operation Desert Storm version) [183], the Keane Combat Scale [193], the Operation Desert Storm Stress Exposure Scale [194], and the Haley Gulf War Combat Exposure Index [35]. With the exception of the Keane Combat Scale (used by Oregon-Washington, Military Health Study, and VA Registry), each instrument was used by only one study. In addition, many studies incorporated self-constructed questions regarding lifetime and combat-related stressors. Thus, creating a framework of direct comparability for this domain may be challenging.

### Behavioral risk factors

All but Kansas I evaluated behavioral risk factors. The most common of these were alcohol and tobacco use. Alcohol use was primarily evaluated using self-constructed questions about current drinking status, alcohol abuse, and number of drinks consumed [2,4,10,12,13,17,25,35,45,51,195,196]. However, Iowa, National Health Survey, and Millennium Cohort used validated instruments. Iowa used the CAGE (Cut back, Annoyance, Guilt, Eye-openers) questionnaire [2,197], and both National Health Survey and Millennium Cohor used the alcohol component of the Patient Health Questionnaire [92].Tobacco use was most commonly queried by asking participants about their smoking status (never, past, or current smoker) [2-4,10,12-14,17,25,35,45,48,139,198]. Additional information obtained varied by study, but included increases or decreases in daily smoking habits [198] and whether participants had smoked at least 100 cigarettes [51,198].

### Validation studies

The studies reviewed herein all gathered self-reported data, which has well-documented limitations, including recall bias and lack of correlation with objective measures. In light of these challenges to data quality, several studies performed reliability and validation analyses, using test-retest methods and comparing self-reported data to clinical evaluations and medical records.

Seabees, Iowa, Millennium Cohort, and Oregon-Washington measured the internal reliability of self-reported survey data, including demographics, physical and functional status, symptoms, health histories, vaccinations, and exposures, using test-retest methods. The results of these studies were mixed, particularly with respect to self-reported exposures, diagnosed health conditions, and symptoms. The Seabees study [31] reported high reliability of demographic attributes (kappas 0.89-1.00), and moderate reliability of exposures (0.60-0.70) and “other” survey items (0.51-0.67). However, self-reported diagnoses and symptoms had widely ranging kappa coefficients of −0.01-1.00 and −0.01-0.86, respectively [31]. The Iowa cohort reported test-retest agreement percentages of 89.6-97.0% and kappa coefficients of 0.39-0.70 for self-reported medical and psychiatric concerns [2]. In the Oregon-Washington evaluation of self-reported exposure reliability, only eight of thirteen exposures had a kappa coefficient statistically greater than 0.4, reflecting poor agreement on the majority of measures [32]. The Oregon-Washington investigators also assessed self-reported exposure misclassification by comparing reported exposure to anthrax and botulinum toxoid vaccines, chemical warfare agents, and pyridostigmine bromide to reported deployment periods, as each exposure was only possible during known periods of time. The results of this comparison suggested that these exposures may be overreported among certain subgroups of veterans [32]. Similarly, an analysis of National Health Survey data found evidence of reporting bias in self-reported anthrax vaccination data [199]. We note that it has been difficult to validate reported exposures due to the absence of objective documentation.

Clinical evaluations and medical records were used to externally validate self-reported information by several studies. As was the case for test-retest reliability analyses, the results were mixed. A New Orleans substudy examined the validity of survey-based PTSD assessments by calculating the agreement between the Structured Clinical Interview for DSM-III-R (SCID) and other PTSD diagnostic measures (agreement 82-100%) [44]. The Seabees validation study, which compared personnel and medical records to survey responses, found kappa coefficients of −0.02-0.48 for self-reported diagnosed illness, 0.41-0.76 for demographic traits, and 0.59-0.66 for “other” characteristics [31]. In the National Health Survey, comparison of reported reasons for clinic visits and hospitalizations to medical records found agreement rates greater than 90% [10,29], and clinical evaluations confirmed an increased risk of certain conditions among deployed veterans [53]. The Iowa study drew on state registries to validate reported birth defects and cancers [60]. In addition, a case validation study in a subset of Iowa cohort members found that only 32% of those who originally reported depressive symptoms met criteria for lifetime depression after a later SCID-IV interview; however, multiple factors may have contributed to this difference [200]. The Millennium Cohort found excellent negative agreement (generally > 95%) and moderate positive agreement when comparing self-reported medical conditions to Department of Defense electronic medical records, suggesting that self-reported data may be useful in excluding the presence of conditions [146]. Finally, in a small-sample comparison of thirteen self-reported medical conditions to medical records, the Devens Cohort Study observed low-moderate kappa coefficients of 0.35-0.64 for most conditions [39]. These heterogeneous findings regarding reliability and validity lend support to concerns about the utility of self-reported data, and emphasize the importance of evaluating reliability and validity early in the study implementation period, and including objective data sources, when resources permit.

## Conclusions

We compared the survey tools used in keystone epidemiologic studies and registries of Gulf War Era veterans, with the intent of highlighting commonalities and differences in efforts aimed at understanding health and risk factors. It is apparent that there are many areas of at least minimal concordance with respect to question and response format. We note that some investigators intentionally drew on the materials used by prior studies, establishing areas of commonality [1,12,60]. Among mental and physical health domains, there was moderate concordance among the measures used to assess medical history, symptoms, functional status, and the diagnoses of chronic fatigue syndrome and fibromyalgia. Most of the exposure assessment instruments also have response formats that can be reduced to the same binary framework. These similarities suggest that meta-analyses of study-level or individual-level data could be performed on several of the subdomains, with varying degrees of loss of detail.

In contrast, there is substantial variation in survey instruments for the subdomains of health status, PTSD, psychological status, psychological trauma, and the diagnoses of fatigue, multiple chemical sensitivity, and multisymptom illness. The nature of this variability differs by subdomain. While this does not preclude the use of meta-analytic techniques, it requires the analyst to carefully consider issues of heterogeneity and whether the instruments demonstrate convergent validity.

We cannot overemphasize the importance of considering the impact of heterogeneity in study design, population, sampling methods, quality, and generalizability to the validity of meta-analyses. While it is possible to carefully combine data from similarly-conducted epidemiologic studies [201], the practical reality is that explicitly accommodating design and quality differences to yield valid inference in analyses of this type remains an extremely difficult (at times impossible) task [202,203]. In addition, restrictions on the sharing of study data may present a logistical challenge to the performance of individual-level joint analyses. This places additional emphasis on the need for investigators to consent future study participants using language that explicitly permits recontact for future studies or (at minimum) sharing of de-identified data under approved protocols for Gulf War Era related research.

Based on our review of the existing studies, we suggest three considerations for future studies of Gulf War Era veterans. First, to engage Gulf War Era research experts during study planning. Second, to gather blood for genetic and proteomic analyses, and link the specimens to survey and medical/administrative records. Third, to carefully consider the strengths and weaknesses of the survey instruments used in the past, and select instruments that are appropriately validated, detailed, and compatible with previous studies. These three activities will support the development of unified data and biospecimen resources with opportunities for analytic collaborations. Through such efforts, epidemiologic research can continue to make important strides that advance our collective ability to enhance the health of these veterans.

## Abbreviations

AF: Air Force; CAGE: Cut back, Annoying, Guilt, Eye-openers; CARC: Chemical agent resistant coating; CFS: Chronic fatigue syndrome; Com: Commissioned officer; DoD: United States Department of Defense; DSM-III-R: Diagnostic and Statistical Manual of Mental Disorders, Third Edition, Revised; FL: Florida; GW: Gulf War; HI: Hawaii; KS: Kansas; MA: Massachusetts; MCS: Multiple chemical sensitivity; MO: Missouri; MRI: Magnetic resonance imaging; MSI: Multisymptom illness; NG: National Guard; Noncom: Noncommissioned officer; OIF: Operation Iraqi Freedom; OMB: Office of Management and Budget; OR: Oregon; PA: Pennsylvania; PTSD: Post-traumatic stress disorder; POW: Prisoner of war; Res: Reserve service; SCID: Structured clinical interview for DSM-III-R; Seabees: Construction Battalion (CB); SF: Medical Outcomes Study Short Form Survey; SW: Southwest; US: United States of America; VA: United States Department of Veterans Affairs; WA: Washington.

## Competing interests

The authors declare that they have no competing interests.

## Authors’ contributions

RBM co-directed the design and execution of the review, wrote the text, and approved the final version. CMT co-directed the design and execution of the review, wrote the text, and approved the final version. SSC contributed to the design of the review, wrote parts of the text, provided critical review, and approved the final version. EH contributed to the design of the review, wrote parts of the text, provided critical review, and approved the final version. GDH contributed to the design of the review, wrote parts of the text, provided critical review, and approved the final version. MJ contributed to the design of the review, wrote parts of the text, provided critical review, and approved the final version. KG contributed to the interpretation of the literature, wrote parts of the text, provided critical review, and approved the final version. TDT wrote part of the text, provided critical review, and approved the final version. DP contributed to the design of the review and the interpretation of the literature, wrote parts of the text, provided critical review, and approved the final version.

## Disclaimer

The contents of this manuscript do not represent the views of the Department of Veterans Affairs or the United States Government.
